# Pre-post changes in physiological, cognitive, and motivational outcomes following an active video game intervention in adolescents and adults: a pilot study

**DOI:** 10.3389/fpubh.2026.1881720

**Published:** 2026-08-12

**Authors:** Ashley Williams, Danielle D. Wadsworth, Thangiah Geetha

**Affiliations:** 1 Department of Food, Nutrition, and Packaging Sciences, Clemson University, Clemson, SC, United States; 2 School of Kinesiology, Auburn University, Auburn, AL, United States

**Keywords:** active video games, adults, body composition, adolescents, family-based intervention, muscular strength, physical activity

## Abstract

**Background:**

Active video games (AVG) are emerging as a promising home-based strategy to increase physical activity by integrating movement into interactive gameplay. However, evidence remains limited regarding their effectiveness across physiological, cognitive, and motivational outcomes in adolescents and adults within household settings.

**Methods:**

In this uncontrolled pilot study, participants completed an 8-week home-based intervention using *Ring Fit Adventure* on the Nintendo Switch. Participants were instructed to engage in at least 60 min of gameplay per week. Pre- and post-intervention assessments included body composition measured by bioelectrical impedance analysis, handgrip strength, and cognitive and fitness performance using the Sway application (balance, reaction time, and executive function tasks). Psychological and motivational constructs were assessed using Self-Determination Theory-based questionnaires. Paired t-tests and correlation analyses were used to evaluate changes over time and associations between variables.

**Results:**

Adolescents demonstrated significant changes in body composition, with increased fat-free mass and reduced fat mass (*p* < 0.05) while, no significant body composition changes were observed in adults. Muscular strength remained stable in adolescents; however, adults exhibited a significant decline in left-hand grip strength (*p* = 0.037), which did not remain significant after correction for multiple comparisons. No robust changes were observed in cognitive or balance outcomes after adjustment. Motivational regulation and exercise enjoyment remained stable throughout the intervention, and psychological constructs were not significantly associated with changes in autonomous motivation.

**Conclusion:**

An 8-week low-dose AVG intervention produced modest and heterogeneous effects, with more favorable physiological responses observed in adolescents than adults. However, changes in adolescent body composition likely reflect normal developmental processes and cannot be attributed solely to the intervention. Overall, commercially available AVGs appear insufficient as a standalone intervention for improving broader cognitive, fitness, or motivational outcomes. Future research should employ controlled designs, optimize intervention dose, improve adherence, and incorporate structured progression to enhance effectiveness.

## 1 Introduction

Physical activity (PA) is essential for both children and adults, providing numerous physical, cognitive, and psychosocial benefits that contribute to overall health and well-being (1). In adults, regular PA is associated with reduced risk of noncommunicable diseases, including cardiovascular diseases, obesity, diabetes, and cancer, while also supporting mental health and cognitive function (1, 2). In children and adolescents, PA plays a critical role in bone mineralization, muscular development, motor skill acquisition, and cognitive performance (1, 3, 4). Despite established guidelines recommending regular moderate-to-vigorous PA, global adherence remains low, with approximately 31% of adults and 80% of adolescents failing to meet recommended levels (5, 6).

Insufficient PA contributes to adverse changes in body composition across the lifespan (7). In adults, physical inactivity accelerates the loss of skeletal muscle mass, contributing to sarcopenia, while promoting increases in adiposity, both of which elevate cardiometabolic disease risk and mortality (8). In children, inadequate PA is associated with poorer muscular development, increased adiposity, and reduced motor competence (9, 10). These early-life patterns may persist into adulthood, increasing long-term disease risk (11). Due to muscle mass and fat mass independently predict long-term health outcomes, interventions that support healthy body composition in both children and adults are critically important.

The home environment represents a powerful context for shaping PA behaviors. Family-level norms, parental modeling, and shared activities strongly influence children’s movement patterns and long-term health trajectories (12). Interventions that engage both children and adults simultaneously may enhance adherence, strengthen social support, and foster sustainable behavior change (13). Active video games (AVGs) offer a promising opportunity for home-based interventions by integrating physical movement into interactive gameplay and transforming traditional sedentary screen time into light-to-moderate physical activity (14–16). Commercially available platforms such as *Ring Fit Adventure* and Wii Fit have demonstrated increased energy expenditure compared to sedentary gaming, suggesting their potential as accessible home-based PA modalities (17, 18).

Beyond acute increases in energy expenditure, structured AVG interventions have demonstrated meaningful physiological adaptations across diverse populations. In children with overweight or obesity, AVG participation has been associated with significant improvements in muscular strength, jump performance, motor competence, and reductions in sedentary time (19). Improvements in motor skills have also been positively associated with increases in vigorous physical activity and muscular power, highlighting the potential benefits for AVGs to influence both behavioral and neuromuscular development (19). In older adults, AVG-based interventions have demonstrated improvements in body mass index (BMI), pulmonary function, blood pressure, balance performance, and spatial cognition, suggesting cardiometabolic and neurocognitive benefits in aging populations (20). Collectively, these findings support that AVGs may influence multiple domains of health across distinct developmental stages.

Despite growing interest in AVGs, several gaps remain in the literature. Most studies have examined single age groups or targeted specific clinical populations, limiting our understanding of how AVG interventions may differentially impact adolescents and adults within the same home-based setting. Furthermore, few studies have simultaneously assessed both physiological and cognitive outcomes across age groups. Given developmental differences in neuromuscular capacity, motivation, and cognitive maturation, responses to AVG interventions may vary substantially between adolescents and adults. Direct comparisons within a shared household setting are needed to better characterize variability in outcomes and engagement patterns across age groups.

Self-Determination Theory (SDT) provides a well-established framework for understanding motivation in PA contexts by distinguishing between autonomous and controlled forms of behavioral regulation and emphasizing the role of basic psychological needs (autonomy, competence, and relatedness) in sustaining engagement (21). In the context of AVG, SDT is particularly relevant because game-based environments may support these psychological needs through autonomy in gameplay choices, competence through skill progression, and relatedness through co-participation within a shared household environment (22). Accordingly, SDT was used in this study to examine whether participation in a home-based AVG intervention was associated with changes in motivational regulation, psychological need satisfaction, and exercise enjoyment across adolescents and adults.

Therefore, the purpose of this study was to examine the effects of an 8-week AVG intervention on body composition, muscular strength, cognitive and fitness performance, and psychological and motivational outcomes in both adolescents and adults. An 8-week intervention duration was selected based on prior pilot exergaming studies demonstrating that short-term (6–8 week) protocols are sufficient to evaluate feasibility and detect preliminary physiological adaptations, including changes in body composition and muscular strength (23). By evaluating multidimensional outcomes within a shared home-based household setting, this study aims to advance the evidence base supporting technology-driven, home-based approaches to increasing physical activity and improving health across developmental stages.

## 2 Materials and methods

### 2.1 Study design

This study was approved by the Auburn University (IRB00000159) and Clemson University (IRB00000481) Institutional Review Board for Research Involving Human Subjects and was conducted in accordance with the Declaration of Helsinki. All participants provided written informed consent prior to participation. The assent from the adolescent was also obtained. Adults completed the Physical Activity Readiness Questionnaire (PAR-Q), while adolescents completed the Physical Activity Readiness Questionnaire for Children (PAR-QC). Only participants who responded “no” to all PAR-Q and PAR-QC items, indicating a low risk for participation in PA, were eligible for inclusion. This study was designed as an 8-week feasibility-oriented intervention based on prior pilot work in active video game research demonstrating that short-term protocols are appropriate for assessing preliminary physiological and behavioral responses.

### 2.2 Participants

Participants were recruited from the local community using a convenience sampling approach that included word of mouth, email, flyers, and social media advertisements. Eligible adult participants were required to be at least 18 years of age, have at least one child aged 10–18 years living in the household, and own a Nintendo Switch gaming system. The participant’s needs to be classified as low risk for participation in PA based on PAR-Q (adults) or PAR-QC (adolescents) screening. Pregnant individuals were excluded from participation. The adolescent age range (10–18 years) was selected to align with the manufacturer-recommended minimum age for *Ring Fit Adventure* (≥10 years) and to capture variability across early to late adolescence.

**Figure 1:**
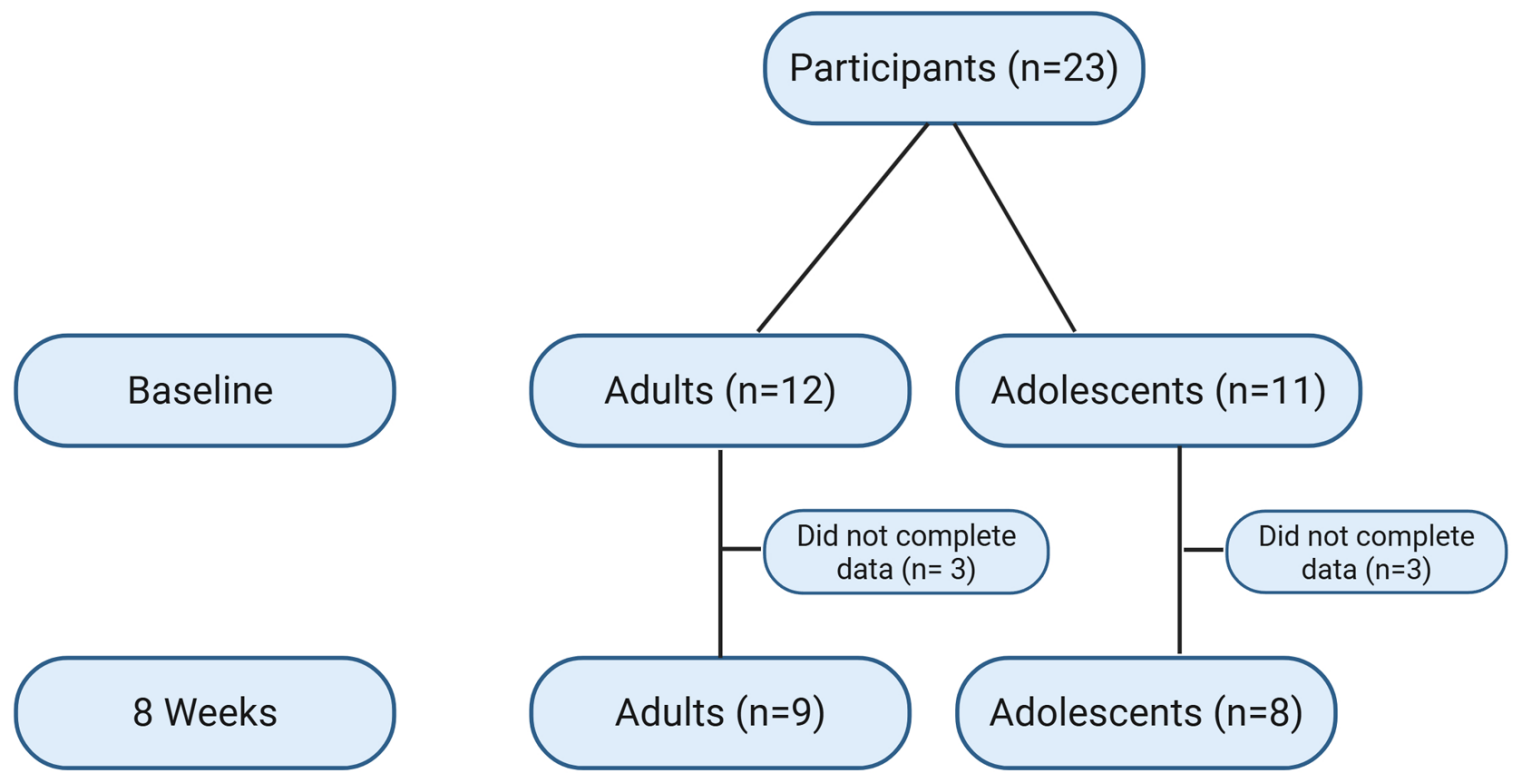
Flow chart of participants through the study. Created with BioRender.com.

A total of 23 participants (12 adults and 11 adolescents) were initially screened, met eligibility criteria, and provided informed consent. Six participants (3 adults and 3 adolescents) withdrew from the study due to incomplete data collection. Participant flow throughout the study is presented in Figure 1.

### 2.3 Measurements

#### 2.3.1 General procedures

All assessments were conducted at Auburn University in a controlled laboratory setting during pre- and post- intervention sessions. Participants were instructed to wear comfortable clothing and to remove shoes and socks prior to anthropometric and body composition measurements to ensure standardization across testing sessions.

#### 2.3.2 Demographics and questionnaires

Participants completed a baseline questionnaire assessing demographic characteristics, including date of birth, race/ethnicity, sex, and handedness. To assess motivational and psychological constructs related to PA, participants completed the Behavioral Regulation in Exercise Questionnaire (BREQ-3), a validated instrument grounded in Self-Determination Theory (SDT) that evaluates multiple forms of behavioral regulation (24–26). At post-intervention, participants completed the BREQ-3 again, in addition to the Basic Psychological Needs in Games Scale and the Exercise Enjoyment Scale. These additional instruments were used to assess psychological needs satisfaction, social relatedness, and overall enjoyment of the AVG intervention (27–29).

Internal consistency reliability for questionnaire measures was assessed using Cronbach’s alpha coefficients. The BREQ-3 demonstrated excellent internal consistency at both pre-intervention (*α* = 0.90) and post-intervention (*α* = 0.92). The Exercise Enjoyment also demonstrated excellent reliability (*α* = 0.94). For the Basic Psychological Needs in Games Scale, autonomy demonstrated lower internal consistency (*α* = 0.55), whereas competence (*α* = 0.81), and relatedness (*α* = 0.77) demonstrated acceptable-to-good reliability. Negatively worded competence items were reverse scored prior to analysis.

#### 2.3.3 Anthropometrics

Standing height was measured to the nearest 0.1 cm, and body weight was measured to the nearest 0.1 kg using a digital scale with a stadiometer (SECA 703). Fat mass (FM) (kg) and fat-free mass (FFM) (kg) were assessed using a bioelectrical impedance analysis (BIA; Tanita, Model TBF-400GS), which has demonstrated validity for assessing body composition in both adults and adolescents (30). To minimize measurement variability, all assessments were conducted at the same time of day for each participant across pre- and post-intervention visits, and participants were instructed to refrain from eating for at least 2 h prior to testing. BMI was calculated as weight (kg) divided by height squared (m^2^) for adults. BMI *z*-score were calculated using a standardized BMI-for-age calculator based on age- and sex-specific reference values for adolescents (31).

#### 2.3.4 Muscular strength

Muscular strength was assessed using a handgrip dynamometer (CAMRY EH 101), a validated, portable, and non-invasive measure of maximal isometric grip strength (32). The dynamometer was adjusted to each participant’s hand size to ensure proper fit. Participants were instructed to stand upright and exert maximal effort for 3 s while holding the dynamometer with the elbow flexed at approximately 90°, with the non-testing arm relaxed at the side. Three trials were completed for each hand, with adequate rest between trials. The highest values for the right and left hands were retained for analysis.

#### 2.3.5 Fitness and cognitive assessments via the sway application

Participants completed physical fitness and cognitive assessments using the Sway medical application (33) on a study-provided mobile device. Each participant was assigned a unique identification code to ensure secure and individualized data collection. All assessments were administered in a standardized manner by the trained study personnel. The following measures were included:

1. The Modified Balance Error Scoring System (mBESS) was used to assess postural stability across five stances performed with eyes closed. During each 10-s trial, participants held the mobile device against their chest. A chair was positioned nearby for safety, although participants were instructed not to use it unless necessary (34).
2. The CDC 30- Second Chair Stand test was used to assess lower- body strength and muscular endurance. Participants completed as many sit-to-stand repetitions as possible within 30 s, beginning and ending in a seated position in a standard chair (35).
3. Reaction Time assessed attention, visual processing, and neuromotor response speed. Participants responded as quickly as possible by tilting the device in response to a visual stimulus (screen color change) (36).
4. Inspection Time assessed attention and visual processing speed. Two T-shaped stimuli were briefly presented, and participants identified which stimulus contained the longer line (37).
5. The Cued Stroop Task assessed attention, cognitive flexibility, and inhibitory control through responses to congruent, neutral, and incongruent color-word stimuli (38).
6. The Go/N-Go (Impulse Control) task assessed response inhibition, sustained attention, and executive control through differential responses to “go” or “no-go” stimuli (39).

Collectively, these assessments provided concurrent measures of physical fitness and cognitive performance relevant to the AVG intervention.

### 2.4 Intervention

Each family received a *Ring Fit Adventure* game (40) and was instructed to participate in the AVG intervention at home for 8 weeks. Participants were asked to complete a minimum of 60 min per week of gameplay, of two 30-min sessions. *Ring Fit Adventure* is an exercise-based action role-playing game developed for the Nintendo Switch system. The game utilizes two primary accessories: the Ring-Con, a Pilates-style resistance ring that holds one Joy-Con controller, and a Leg Strap, which secures the second Joy-Con on the participant’s thigh to capture lower-body movement. The intervention was standardized at the level of game platform and mode, as all participants were instructed to use Adventure Mode exclusively throughout the study period. Each session consisted of a 5-min warm-up, active gameplay, and a 5-min cool-down period. However, exercise intensity and progression were self-directed and depended on participant effort, game responsiveness, and individual gameplay decisions within Adventure Mode. As such, while the dose (time and frequency) and game mode were standardized, the internal exercise stimulus (intensity and specific activities) varied across participants. Therefore, while all participants received the same structured protocol (mode, duration, and frequency), the internal training stimulus varied due to self-selected intensity and progression.

To monitor adherence and quantify exposure, participants submitted weekly email screenshots of the in-game activity summary generated by *Ring Fit Adventure*, which displays cumulative gameplay duration, exercise time, and selected difficulty level. Active exercise time is generated by the game’s accelerometer-based tracking system, which distinguishes active gameplay from total play time based on movement detected by the Nintendo Switch controllers and Ring-Con accessory. Screenshots were reviewed weekly by the research team for completeness and consistency. Extracted values (total gameplay time and active exercise minutes) were independently checked prior to entry into a structured Excel database. Any missing or unclear entries were clarified with participants when necessary. These verified weekly logs were aggregated across the 8-week intervention to derive total and average exposure metrics, which were subsequently used in dose–response analyses examining associations between gameplay exposure and changes in physiological outcomes. The maximum potential dose of the intervention was 480 min across the 8-week period (16 sessions x 30 min).

Although participants were recruited as family units, the intervention did not require or structure synchronous or co-participatory gameplay between adolescents and adults. Each participant completed gameplay individually within the home environment at self-selected times. Adults and adolescents followed the same general protocol but did not engage in mandated co-play, turn-taking, or joint sessions. Adult participants may have provided informal encouragement or general oversight; however, these interactions were neither standardized nor measured, and no structured family interaction component was incorporated into the intervention

### 2.5 Statistical analysis

All statistical analyses were performed using R (version 4.5.1). Descriptive statistics (mean, standard deviation, and standard error of the mean) were calculated for participant characteristics and baseline variables, including age, BMI, and BMI z-score. Baseline characteristics were compared between participants who completed the intervention (completers) and those who withdrew (dropouts) to assess potential attrition bias. Welch’s independent samples t-tests were used for normally distributed variables, whereas Wilcoxon rank-sum tests were performed as sensitivity analyses when normality assumptions were not met. Variables examined included body weight, BMI, FM, FFM, and grip strength.

Internal consistency reliability for questionnaire-based measures was assessed using Cronbach’s alpha coefficients. Paired-samples t-tests were used to examine pre-post intervention differences in body composition, muscular strength, and fitness and cognitive outcomes assessed via the Sway application. Normality assumptions were evaluated using Shapiro Wilk tests, and Wilcoxon signed-rank tests were performed as non-parametric sensitivity analyses when appropriate. Effect sizes for paired comparisons were calculated using Hedges’ g to account for the small sample size. Bonferroni-adjusted *p*-values were applied to muscular strength and cognitive/balance outcomes to account for multiple comparisons, with statistical significance defined as an adjusted *p* < 0.05. Psychological outcomes included motivation (Relative Autonomy Index; RAI), motivational regulation subscales (amotivation, external regulation, introjected regulation, identified regulation, integrated regulation, and intrinsic motivation), basic psychological needs (autonomy, competence, and relatedness), and exercise enjoyment. RAI_post was defined as the post-intervention Relative Autonomy Index score representing motivational status following the intervention. RAI_change was calculated as the difference between post- and pre- intervention scores (RAI_post – RAI_pre) and treated as an exploratory measure of within-person change. Subscale scores were computed as the means of their respective items. Paired-samples t-tests were used to evaluate pre–post changes in RAI and motivational regulation subscales, with Wilcoxon signed-rank tests performed as sensitivity analyses where appropriate. Cohen’s d for paired samples was used to estimate effect sizes for psychological outcomes specific to the BREQ.

Dose–response relationships between physical activity exposure (active minutes per week) and changes in physiological outcomes (fat mass, fat-free mass, and grip strength) were examined using Pearson correlation analyses. Associations between psychological constructs (autonomy, competence, relatedness, and enjoyment) and motivational outcomes (RAI_post and RAI_change) were evaluated using univariate Pearson correlation analyses. Statistical significance was set at *p* < 0.05. Where applicable, both unadjusted and Bonferroni- adjusted *p*-values are reported. Given the exploratory nature of this pilot study and the limited sample size, findings were interpreted cautiously, with emphasis placed on effect sizes, consistency across parametric and non-parametric tests, and magnitude of associations rather than statistical significance alone.

## 3 Results

A total of 23 participants were initially enrolled in the study, of which 17 (9 adults and 8 adolescents) completed the 8-week intervention, yielding a retention rate of 74% (6 dropouts; 26%). On average, adolescents participated in 55 min of gameplay per week, of which 42 min consisted of active in-game exercise. Adults participated in an average of 22 min of gameplay per week, including 11 min of active exercise.

### 3.1 Participant flow and baseline comparisons

Baseline characteristics were compared between participants who completed the study and those who withdrew to assess potential attrition bias (Supplementary Table S1). No significant differences were observed in weight, BMI, FM, or FFM (all *p* > 0.05). However, participants who withdrew demonstrated significantly higher baseline grip strength for both the right (*p* = 0.004) and left hand (*p* = 0.002), suggesting potential baseline difference in muscular strength between completers and non-completers (dropout) participants.

### 3.2 Baseline and post-intervention characteristics

Pre- and post- intervention demographic and anthropometric characteristics for adolescents and adults are presented in Table 1. Among adolescents, baseline mean age was 14.65 ± 2.41 years. Mean body weight increased slightly from 46.8 ± 17.8 kg to 48.1 ± 16.6 kg (*p* = 0.226), while height increased from 61.84 ± 4.89 into 62.33 ± 4.97 in (*p* = 0.103). Among adults, the mean age was 45.67 ± 2.74 years. Body weight increased slightly from 84.9 ± 26.9 kg to 85.9 ± 28.3 kg (*p* = 0.183), while height remained stable (66.47 ± 3.21 inches pre-intervention vs. 66.80 ± 3.33 inches post-intervention, *p* = 0.356). No significant changes were observed in anthropometric variables for either group across the intervention period.

**Table 1:** Pre- and post-intervention demographic and anthropometric characteristics.

Parameter	Pre (Week 0)	Post (Week 8)	*p*-value
Adolescents			
Total participants	8	8	–
Sex (Female/Male)	1/7	1/7	–
Race (Asian/White)	4/4	4/4	–
Age (years)	14 ± 2.83	14 ± 2.83	–
Weight (kg)	46.8 ± 17.8	48.1 ± 16.6	0.226
Height (inches)	61.84 ± 4.89	62.33 ± 4.97	0.103
Adults			
Total participants	9	9	–
Sex (Female/Male)	4/5	4/5	–
Race (Asian/White)	4/5	4/5	–
Age (years)	45.67 ± 2.74	46.14 ± 2.76	–
Weight (kg)	84.9 ± 26.9	85.9 ± 28.3	0.183
Height (inches)	66.47 ± 3.21	66.80 ± 3.33	0.356

BMI, Body Mass Index. Data presented as mean ± standard deviation.

### 3.3 Body composition outcomes

**Figure 2:**
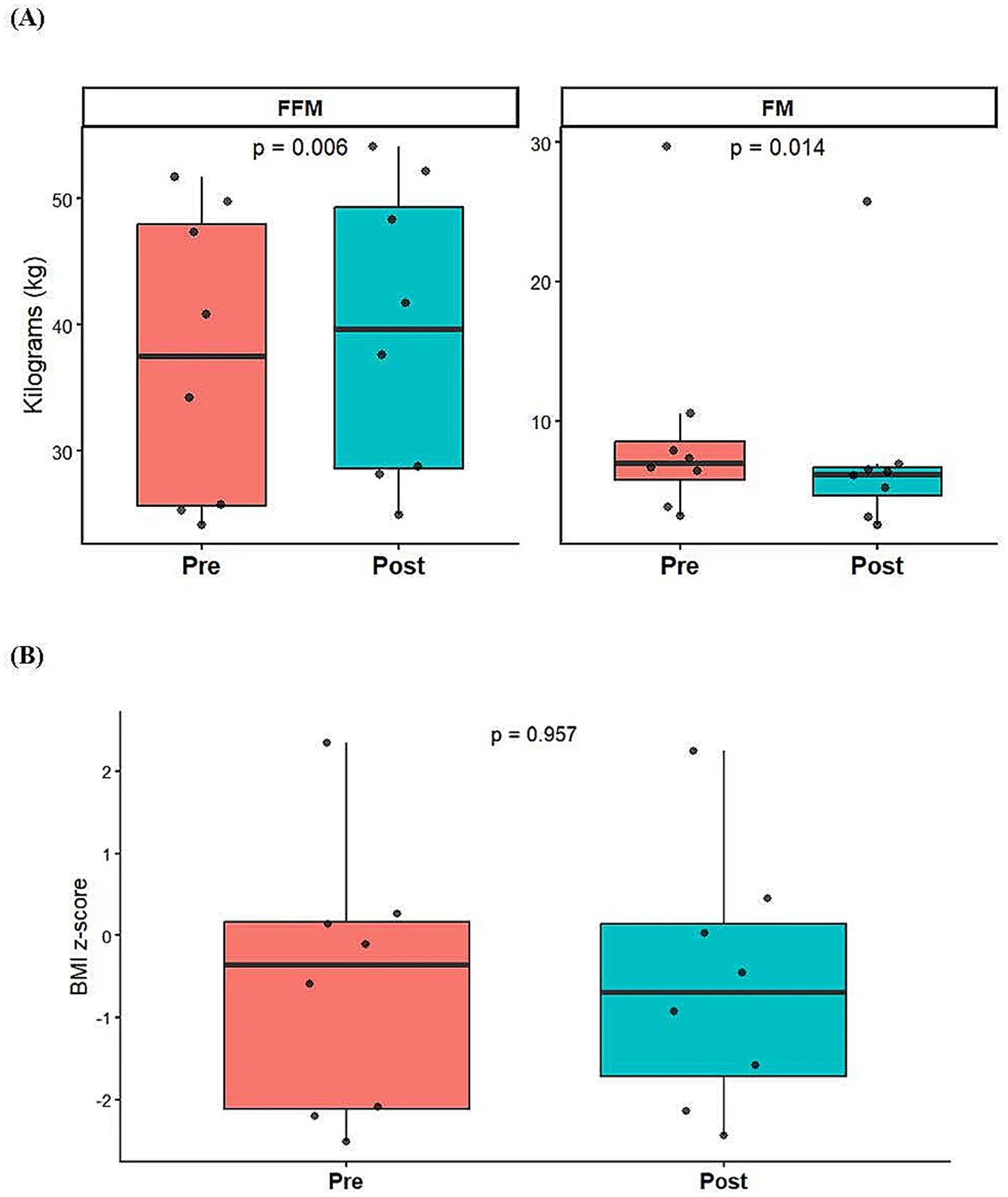
Adolescents’ body composition pre- and post-intervention. **(A)** Fat-free mass (FFM) and fat mass (FM); **(B)** BMI z-score. Data are presented as mean ± SD. *p-values are shown for pre–post comparison.

Among adolescents, FFM increased significantly from 37.46 ± 11.59 kg to 38.50 ± 11.21 kg (*p* = 0.006), with a mean change of 1.04 kg (95% Cl: 0.84 to 3.35 kg). FM decreased significantly from 9.45 ± 8.50 kg to 8.75 ± 8.31 kg (*p* = 0.014), with a mean change of −0.70 kg (95% Cl: −3.61 to −0.07 kg). (Figure 2A). BMI z-score did not change significantly over the intervention period (Figure 2B).

**Figure 3:**
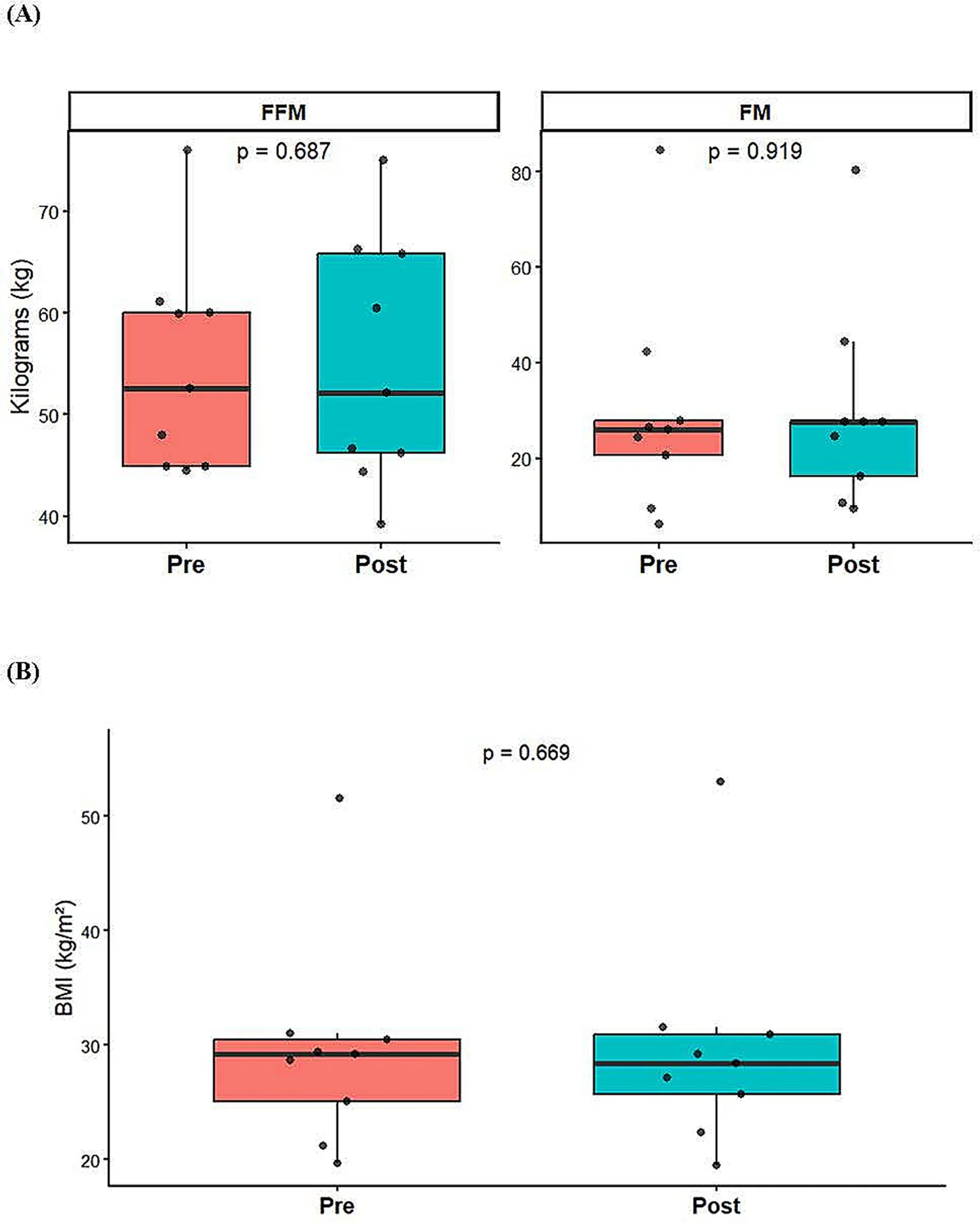
Adults body composition pre- and post-intervention. **(A)** Fat-free mass (FFM) and fat mass (FM), **(B)** BMI. Data are presented as mean ± SD. * *p*-values are shown for pre–post comparison.

Among adults, no significant changes in body composition were observed. FFM remained stable from 54.75 ± 10.60 kg to 54.30 ± 11.01 kg (*p* = 0.687), with a mean change of −0.45 kg (95% Cl: −4.72 to 3.82 kg). FM showed minimal change from 29.74 ± 23.13 kg to 29.89 ± 22.55 kg (*p* = 0.919), with a mean change of 0.15 kg (95% CI: −4.67 to 5.12 kg) (Figure 3A). BMI also remained unchanged (pre: 29.59 ± 9.17 kg/m^2^ vs. post: 29.74 ± 9.55 kg/m^2^; *p* = 0.669; Figure 3B). These findings were consistent across both paired t-tests and Wilcoxon signed- rank tests, indicating robustness of the results. Across both groups, changes in FM and FFM were not always fully concordant with changes in body weight, reflecting expected variability in BIA body composition estimates.

### 3.4 Muscular strength outcomes

Handgrip strength was assessed bilaterally in adolescents and adults at pre- and post-intervention (Table 2). Among adolescents, no significant changes were observed in either the right-hand grip strength (pre: 20.9 ± 7.94 kg; post: 20.6 ± 7.05 kg; *p* = 0.780) or the left-hand grip strength (pre: 18.2 ± 5.90 kg; post: 18.8 ± 5.67 kg; *p* = 0.419). Effect sizes were small (Hedges’ g = −0.093 for the right hand; g = 0.274 for the left hand), indicating minimal practical change.

**Table 2:** Pre- and post-intervention muscular strength in adolescents and adults.

Group	Hand	Pre (kg) (Week 0)	Post (kg) (Week 8)	Effect size	*p*-value	Adjusted *p*-value
Adolescents	Right	20.9 ± 7.94	20.6 ± 7.05	−0.093	0.780	1.000
Adolescents	Left	18.2 ± 5.90	18.8 ± 5.67	0.274	0.419	1.000
Adults	Right	37.9 ± 11.5	36.4 ± 11.0	−0.453	0.176	0.702
Adults	Left	35.6 ± 12.5	32.6 ± 12.6	−0.763	**0.037**	0.147

Values are presented as mean ± standard deviation (kg). Effect sizes represent the standardized change from pre (Week 0) to post (Week 8). Bonferroni- adjusted p-values were used, with significance defined as *p* < 0.05. Significant values presented in bold.

Among adults, right-hand grip strength decreased slightly from 37.9 ± 11.5 kg to 36.4 ± 11.0 kg, although this change was not statistically significant (*p* = 0.176). In contrast, left-hand grip strength declined significantly from 35.6 ± 12.5 kg to 32.6 ± 12.6 kg (*p* = 0.037), with a large effect size (Hedges’ g = −0.763). However, after applying a Bonferroni correction for multiple comparisons, this finding was no longer statistically significant (adjusted *p* = 0.148).

These findings were consistent across both paired t-tests and Wilcoxon signed- rank tests, indicating robustness of the results. Overall, the intervention was not associated with meaningful changes in muscular strength in adolescents, while adults demonstrated no statistically robust changes following adjustment for multiple comparisons.

### 3.5 Fitness and cognitive assessments outcomes

Cognitive and balance outcomes were assessed in adolescents and adults at pre- and post-intervention (Table 3). Among adolescents, no cognitive or balance outcomes demonstrated statistically significant changes following Bonferroni correction for multiple comparisons. Although inspection time differed between pre- and post-intervention (pre: 69.00 ± 30.87 *ms*; post: 43.88 ± 26.70 *ms*; *p* = 0.017), this finding was no longer statistically significant after adjustment (adjusted *p* = 0.330).

**Table 3:** Pre- and post-intervention cognitive and balance outcomes in adolescents and adults.

Parameter	Pre (Week 0)	Post (Week 8)	*p*-value	Adjusted *p*-value
Adolescents				
Cued Stroop task	67.77 ± 33.61	72.62 ± 33.61	0.707	1.000
Feet together eyes closed	92.52 ± 7.51	79.89 ± 32.95	0.213	1.000
Impulse control (ms)	429.00 ± 86.45	352.13 ± 154.83	0.207	1.000
Inspection time (ms)	69.00 ± 30.87	43.88 ± 26.70	**0.0165**	0.330
Simple reaction time (ms)	260.63 ± 65.46	205.25 ± 86.63	0.206	1.000
Single leg eyes closed left	49.62 ± 37.14	49.82 ± 42.20	0.951	1.000
Single leg eyes closed right	73.44 ± 34.20	52.40 ± 34.73	0.312	1.000
Sit to stand	11.75 ± 5.50	12.13 ± 8.36	0.886	1.000
Tandem eyes closed left	78.83 ± 16.10	66.12 ± 35.08	0.321	1.000
Tandem eyes closed right	70.23 ± 25.34	76.16 ± 34.73	0.661	1.000
Adults				
Cued Stroop task	81.01 ± 11.93	80.45 ± 16.93	0.914	1.000
Feet together eyes closed	97.87 ± 2.09	97.87 ± 2.33	0.655	1.000
Impulse control (ms)	377.67 ± 50.99	390.11 ± 79.48	0.479	1.000
Inspection time (ms)	41.00 ± 32.52	44.11 ± 31.51	0.761	1.000
Simple reaction time (ms)	262.11 ± 25.91	252.33 ± 26.19	0.329	1.000
Single leg eyes closed left	73.12 ± 23.32	62.29 ± 19.38	0.173	1.000
Single leg eyes closed right	57.92 ± 31.21	70.54 ± 16.72	0.311	1.000
Sit to stand	11.00 ± 3.00	10.22 ± 6.24	0.65	1.000
Tandem eyes closed left	89.64 ± 11.55	87.32 ± 16.64	0.686	1.000
Tandem eyes closed right	93.70 ± 6.19	90.21 ± 6.93	**0.00867**	0.173

Values are presented as mean ± standard deviation (SD). Bonferroni-adjusted p-values were used, with significance defined as p_adj < 0.05. ms = milliseconds. Statistically significant results (*p* < 0.05) are in bold.

Similarly, among adults, no cognitive or balance outcomes remained statistically significant after Bonferroni correction. Tandem eyes closed (right) differed between pre- and post-intervention (pre: 93.70 ± 6.19; post: 90.21 ± 6.93; *p* = 0.009), but this finding was not statistically significant after adjustment (adjusted *p* = 0.173).

These findings were consistent across both paired t-tests and Wilcoxon signed-rank tests, indicating robustness of the results. Overall, no statistically significant changes in cognitive or balance outcomes were observed in either adolescents or adults following adjustment for multiple comparisons.

### 3.6 Psychological and motivational outcomes

Participants completed pre- and post-intervention assessments of motivational regulations; however, complete psychological outcome data were available only for 16 participants due to missing responses. Normality of distribution for subscale change scores was assessed using the Shapiro–Wilk test prior to inferential analyses. As shown in Table 4, descriptive analyses of Self-Determination Theory–based motivational constructs indicated no statistically significant pre- to post-intervention changes across motivational regulation subscales.

**Table 4:** Pre-and post-intervention motivational regulations.

Variable	Pre (Week 0)	Post (Week 8)	Effect size	*p*-value
Adolescents				
Amotivation	3 ± 2.62	2.25 ± 3.06	−0.186	0.615
External regulation	6.25 ± 3.92	4.75 ± 3.41	−0.409	0.320
Introjected regulation	5.25 ± 4.13	4.25 ± 3.33	−0.331	0.381
Identified regulation	8.38 ± 4.37	8.62 ± 4.84	0.122	0.741
Integrated regulation	5.88 ± 5.51	6.5 ± 4.57	0.201	0.588
Intrinsic motivation	8.38 ± 4.96	8.38 ± 5.78	0	1
Adults				
Amotivation	1.25 ± 1.75	0.875 ± 1.46	−0.409	0.285
External regulation	3.75 ± 2.60	4.12 ± 4.58	0.0833	0.820
Introjected regulation	7 ± 3.42	5.88 ± 4.39	−0.435	0.259
Identified regulation	10.5 ± 1.85	10.5 ± 2.67	0	1
Integrated regulation	5.5 ± 4.28	5.38 ± 3.42	−0.0560	0.879
Intrinsic motivation	9.12 ± 3.68	9 ± 3.78	−0.150	0.685

Data are presented as mean ± standard deviation (SD). Effect sizes are reported as Cohen’s d for paired samples. Amotivation, external regulation, introjected regulation, identified regulation, integrated regulation, and intrinsic motivation represent subscales of behavioral regulation. Statistical significance was set at *p* < 0.05.

Among adolescents, no significant changes were observed in motivation, external regulation, introjected regulation, identified regulation, integrated regulation, or intrinsic motivation. Although small directional changes were noted in several subscales, including decreases in more controlled forms of motivation and slight increases in more autonomous forms, none reached statistical significance (Table 4). Intrinsic motivation remained stable across time points. Similarly, among adults, no statistically significant changes were observed in any motivational regulation subscale following the intervention. Minor fluctuations were observed across subscales; however, overall motivational profiles remained stable from pre- to post-intervention (Table 4).

As shown in Table 5, univariate Pearson correlation analyses were conducted to examine associations between psychological constructs (autonomy, competence, relatedness, and enjoyment) and motivational outcomes (RAI post-intervention and RAI change). In the combined sample, autonomy, competence, and relatedness were not significantly associated with either RAI outcome. Enjoyment demonstrated a moderate positive association with RAI post-intervention (*r* = 0.73, *p* = 0.0019), which remained significant after Bonferroni correction (adjusted *p* = 0.015). Although enjoyment was also positively associated with RAI change (*r* = 0.55, *p* = 0.033), this association did not remain statistically significant following adjustment for multiple comparisons (adjusted *p* = 0.265).

**Table 5:** Pearson correlations between psychological need satisfaction, enjoyment, and relative autonomy index outcomes in the combined sample (*N* = 17).

Predictor	Outcome	*r*	*p*-value	Adjusted *p*-value
Autonomy	RAI_change	0.104	0.700	1.000
Autonomy	RAI_post	−0.222	0.408	1.000
Competence	RAI_change	0.127	0.640	1.000
Competence	RAI_post	−0.00187	0.995	1.000
Relatedness	RAI_change	0.169	0.531	1.000
Relatedness	RAI_post	−0.102	0.707	1.000
Enjoyment	RAI_change	0.551	0.0332	0.265
Enjoyment	RAI_post	0.734	**0.00185**	**0.0148**

Data are presented for the combined sample of adolescents and adults (*n* = 17). Pearson correlation coefficients (r) describe the univariate association between post-intervention psychological need satisfaction (autonomy, competence, and relatedness), enjoyment, and Relative Autonomy Index (RAI) outcomes. RAI_change represents the change in RAI from pre- to post-intervention, and RAI_post represents post-intervention RAI. Adjusted *p*-values were calculated using the Bonferroni correction for multiple comparisons. Bold values indicate statistically significant differences (*p* < 0.05).

Exploratory group-stratified correlation analyses are presented in Supplementary Table S2. Similar patterns were observed within adolescents and adults; however, no consistent statistically significant associations were identified after adjustment for multiple comparisons. Given the small subgroup sample sizes, these exploratory analyses should be interpreted with caution and are intended to provide descriptive context rather than definitive evidence of group-specific relationships.

### 3.7 Dose- response associations between active gameplay and physiological outcomes

Dose–response relationships between active gameplay exposure and physiological outcomes were examined using univariate Pearson correlations. As shown in Supplementary Table S3, associations between active gameplay (average weekly minutes during the 8-week intervention) and changes in fat mass, fat-free mass, and grip strength were generally weak and not statistically significant after correction for multiple comparisons.

Correlation coefficients ranged from *r* = −0.27 to 0.37. The strongest (non-significant) associations were observed for total grip strength (*r* = 0.37) and left-hand grip strength (*r* = 0.34). Scatterplots illustrating these relationships are provided in Supplementary Figure S1, demonstrating weak trends and substantial inter-individual variability.

## 4 Discussion

The present uncontrolled pilot study examined the effects of an 8-week home-based AVG intervention on body composition, muscular strength, cognitive and fitness performance, and motivational outcomes in adolescents and adults. Overall, the findings suggest that a low-dose, commercially available AVG delivered in a naturalistic home environment produces modest and heterogeneous effects, with more observable physiological adaptations in adolescents than in adults. This pattern aligns with prior evidence suggesting that exergaming can elicit light-to-moderate PA but produces inconsistent health outcomes when implemented without structured progression or supervision (41).

A key finding among adolescents was the observation of favorable changes in body composition, characterized by increases in FFM and reductions in FM. Notably, BMI z-scores remained stable, consistent with evidence that BMI is a less sensitive indicator of early compositional changes in youth compared with direct measures of FM and FFM (42, 43). This dissociation between BMI and body composition aligns with prior pediatric literature demonstrating that compartment-specific measures provide greater sensitivity for detecting early physiological change (43). The observed improvements may be attributable to increased light-to-moderate PA embedded within gameplay, which has been previously shown to elevate energy expenditure compared to sedentary screen-based behaviors (42). It is also important to acknowledge that body composition was assessed using bioelectrical impedance analysis, which is sensitive to hydration status and other short-term physiological factors and may therefore have contributed to variability in FM and FFM estimates.

In contrast, adults did not demonstrate meaningful changes in body composition, possibly due to lower adherence and insufficient cumulative activity exposure. This finding is consistent with evidence that exergaming engagement declines with age in free-living environments (44). However, given the uncontrolled design and small sample size, these interpretations remain speculative. Muscular strength outcomes remained stable in adolescents, with no significant differences observed in handgrip strength. Among adults, right-hand grip strength showed a small non-significant decline, while left-hand grip strength decreased at the unadjusted level but was not statistically significant after correction for multiple comparisons. The isolated left-hand finding may be interpreted as a chance finding or measurement variability rather than a true physiological decline. Given the relatively low weekly participation time reported in adults, these findings may reflect insufficient training stimulus to maintain baseline strength levels rather than a direct negative effect of the intervention. Similar conclusions have been reported in exergaming studies showing limited or inconsistent improvements in muscular strength unless intervention incorporates structured resistance components (45, 46).

Cognitive and balance outcomes assessed via the Sway application revealed no robust intervention effects. After adjustment for multiple comparisons, no cognitive or balance outcome showed a significant change. These outcomes included measures of executive function (cued Stroop task, impulse control, inspection time, and simple reaction time) as well as balance-related performance (feet together eyes closed, single-leg stance, sit-to-stand, and tandem stance tasks) in both adolescents and adults. In addition, given the repeated administration of identical cognitive and balance tasks, practice effects may have contributed to any observed pre-post differences at the unadjusted level. These findings differ from previous studies reporting cognitive and executive function benefits following structured exergaming interventions in youth populations (47, 48). However, these benefits are typically observed under highly controlled laboratory or school-based protocols with structured cognitive-motor demands, whereas the present study reflects real-world, self-directed gameplay. Similarly, improvements in balance and motor control have been reported in clinical and older adult populations, these effects are typically observed in rehabilitative contexts with targeted training rather than commercial gaming environments (49, 50). Collectively, these findings suggest that cognitive and motor transfer effects are not inherent to AVGs but depend strongly on intervention specificity and dose.

From a behavioral and psychological perspective, motivational regulation remained stable across the intervention period in both adolescents and adults. No significant changes were observed in self-determination theory–based constructs, and psychological need satisfaction did not predict changes in Relative Autonomy Index outcomes. These findings suggest that short-term exposure to a single AVG title may be insufficient to meaningfully shift deeper motivational regulation processes (51). Although theoretical frameworks of self-determination propose that autonomy-supportive and engaging environments may enhance intrinsic motivation, the present findings indicate that such effects are not guaranteed in real-world, minimally structured implementations (52).

A further critical observation is the marked disparity in participation between adolescents and adults, with adolescents accumulating substantially greater gameplay and exercise exposure. This difference introduces a major methodological limitation, as adherence differed so greatly between adolescents and adults that age-group effects cannot be disentangled from dose-related effects. This heterogeneity in dose exposure likely attenuates intervention effects in adults and highlights a persistent translational challenge in family-based digital health interventions: shared interventions do not necessarily produce shared behavioral engagement (12, 53, 54). The adolescent sample also spanned a broad developmental range (10–18 years), which may have introduced additional heterogeneity in physical maturation and engagement.

Importantly, although the intervention followed a standardized protocol (Adventure Mode, prescribed 30-min sessions, and a minimum weekly dose), implementation remained inherently variable. Gameplay was self-paced and unsupervised, meaning that intensity, progression through game levels, effort, and in-game strategy likely differed across participants. This heterogeneity increases ecological validity but reduces standardization of the stimulus and likely contributed to variability in observed outcomes and weak dose–response relationships.

Consistent with this interpretation, dose–response analyses demonstrated generally weak and non-significant associations between gameplay exposure and changes in physiological outcomes after correction for multiple comparisons. Although modest positive trends were observed for grip strength variables, these associations were inconsistent and not robust. Scatterplot visualizations further highlighted substantial inter-individual variability, reinforcing the absence of a clear dose–response relationship within this sample. Collectively, these findings suggest that commercially available AVGs should not be viewed as universally effective stand-alone interventions but rather as context-dependent tools whose effectiveness appears to depend on participant engagement, adherence, and intervention dose (41, 42, 45, 52). Within the constraints of this uncontrolled pilot study, adolescents demonstrated modest favorable changes in body composition, whereas cognitive, fitness, and motivational outcomes remained largely unchanged across both age groups. Given the uncontrolled design, small convenience sample, and substantial variability in adherence, these findings should be considered preliminary and hypothesis-generating rather than confirmatory. Future randomized controlled trials with larger samples, improved adherence strategies, and standardized monitoring of exercise intensity are needed to clarify the physiological and behavioral effects of home-based AVG interventions.

## 5 Limitations and future directions

This study has several limitations. First, dietary intake was not assessed, which may have independently influenced body composition outcomes and limits attribution of observed changes to the intervention. Second, the absence of a control group limits causal inference and limits the ability to distinguish intervention effects from normal developmental or environmental changes. The study also used a convenience sample with a small number of completers (n = 17), resulting in limited statistical power and an increased risk of type II error. Sensitivity analysis indicated that, given the current sample size, only large effect sizes (approximately d ≥ 0.8) could be reliably detected at *α* = 0.05, further emphasizing the exploratory nature of the findings. In addition, the study was not randomized, and neither outcome assessors nor analysts were blinded, which may have introduced potential bias.

Adherence differed between adolescents and adults, with lower participants in adults likely attenuating intervention effects. This imbalance limits the ability to isolate age-related effects from dose-related effects and reduces interpretability of between-group comparisons. Intervention exposure was therefore not equivalent across groups, which may have confounded observed outcomes. Gameplay was also self-paced and unsupervised, introducing variability in intensity and progression that is not captured by total duration alone. Although adherence monitoring included gameplay screenshots that documented activity minutes generated by the *Ring Fit Adventure* accelerometer-based tracking system, these measures could not verify physiological effort (e.g., heart rate or perceived exertion) or completely rule out participant noncompliance or intentional manipulation. Moreover, the adolescent sample (10–18 years) spanned a wide developmental range, which may have introduced additional heterogeneity in physical maturation and engagement.

Body composition was assessed using BIA. Although this has demonstrated validity or assessing body composition in both adults and adolescents (30), it is sensitive to hydration status, recent physical activity, and dietary intake. Finally, the use of a single commercial AVG and the relatively short duration and low prescribed dose may have been insufficient to elicit consistent physiological and cognitive adaptations. The exploratory design and multiple outcomes also increase the risk of type I error.

Future studies should employ randomized controlled designs with larger and more diverse samples. Incorporating objective measures of physical activity (e.g., accelerometry) and dietary intake should be incorporated to improve behavioral characterization. More precise body composition methods, such as dual- energy X-ray absorptiometry, or standardized hydration-controlled assessment protocols should also be considered to improve measurement accuracy. In addition, future interventions should include structured and progressive training, objective monitoring for exercise intensity and physiological load, and strategies to enhance adherence, particularly among adults. Studies using multiple AVG platforms, longer intervention periods, and more standardized gameplay progression may further clarify the potential role of AVGs in promoting health across age groups.

## 6 Conclusion

This uncontrolled pilot study provides preliminary evidence that an 8-week home-based AVG intervention yields modest and domain-specific effects. Small changes in body composition were observed in adolescents, while changes in muscular strength, cognitive performance, and motivation were limited and inconsistent across both adolescents and adults. Lower engagement among adults likely attenuated intervention exposure and may have contributed to between-group differences in observed outcomes. Overall, commercially available active video games may serve as a feasible and engaging approach for increasing physical activity in home environments, however, their effectiveness as a standalone strategy for producing comprehensive health benefits appears limited. Future research should focus on optimizing intervention dose, improving adherence across age groups, and incorporating more structured and progressive training elements to better evaluate the health impact of AVG based interventions.

## Data Availability

The original contributions presented in the study are included in the article/Supplementary material, further inquiries can be directed to the corresponding author/s.
