# Supplementary material for: Pre-post changes in physiological, cognitive, and motivational outcomes following an active video game intervention in adolescents and adults: a pilot study
Source: Front Public Health. 2026 Aug 12;14:1881720. doi: 10.3389/fpubh.2026.1881720 (PMC13506757; doi:10.3389/fpubh.2026.1881720)
Supplement: Supplementary file 1 [file Supplementary_file_1.docx]

**Supplementary Table S1.** Baseline Characteristics of Completers and Dropouts

| **Variable** | **Completers (n=17)** | **Dropouts (n=6)** | ***p*-value** |
| --- | --- | --- | --- |
| Weight (kg) | 66.97 ± 29.77 | 72.68 ± 45.19 | 0.782 |
| BMI (kg/m²) | 24.61 ± 9.19 | 25.28 ± 13.10 | 0.911 |
| Fat Mass (kg) | 20.16 ± 20.17 | 20.67 ± 20.57 | 0.959 |
| Fat-Free Mass (kg) | 46.52 ± 13.91 | 51.27 ± 25.34 | 0.678 |
| Right Grip Strength (kg) | 29.92 ± 13.08 | 53.72 ± 13.09 | **0.004** |
| Left Grip Strength (kg) | 27.41 ± 13.12 | 49.15 ± 10.67 | **0.002** |

Baseline characteristics were compared between completers (n = 17) and dropouts (n = 6) to assess potential attrition bias. Welch’s independent samples t-tests. When the assumption of normality was not met, Wilcoxon rank-sum tests were conducted as sensitivity analyses; results were consistent across methods. Variables included body weight, body mass index (BMI), fat mass (FM), fat-free mass (FFM), and handgrip strength. Values are presented as mean ± standard deviation. Statistical significance was set at p < 0.05.

**Supplementary Table S2.** Group-Stratified Pearson Correlations Between Psychological Need Satisfaction, Enjoyment, and Relative Autonomy Index Outcomes in Adolescents and Adults

| **Predictor** | **Outcome** | **r** | ***p*-value** | **Adjusted *p*-value** |
| --- | --- | --- | --- | --- |
| **Adolescents** | | | | |
| Autonomy | RAI_change | -0.00537 | 0.990 | 1.000 |
| Autonomy | RAI_post | -0.517 | 0.190 | 1.000 |
| Competence | RAI_change | 0.324 | 0.434 | 1.000 |
| Competence | RAI_post | 0.0802 | 0.850 | 1.000 |
| Relatedness | RAI_change | 0.0230 | 0.957 | 1.000 |
| Relatedness | RAI_post | -0.356 | 0.387 | 1.000 |
| Enjoyment | RAI_change | 0.722 | 0.0433 | 0.346 |
| Enjoyment | RAI_post | 0.782 | 0.0218 | 0.174 |
| **Adults** | | | | |
| Autonomy | RAI_change | 0.324 | 0.433 | 1.000 |
| Autonomy | RAI_post | 0.218 | 0.603 | 1.000 |
| Competence | RAI_change | -0.242 | 0.564 | 1.000 |
| Competence | RAI_post | -0.158 | 0.708 | 1.000 |
| Relatedness | RAI_change | 0.448 | 0.266 | 1.000 |
| Relatedness | RAI_post | 0.514 | 0.193 | 1.000 |
| Enjoyment | RAI_change | 0.269 | 0.560 | 1.000 |
| Enjoyment | RAI_post | 0.652 | 0.112 | 0.900 |

Pearson correlation coefficients (*r*) are presented separately for adolescents (*n* = 8) and adults (*n* = 9). Psychological need satisfaction (autonomy, competence, and relatedness) and enjoyment were assessed following the intervention. RAI_change represents the change in Relative Autonomy Index (RAI) from pre- to post-intervention, and RAI_post represents post-intervention RAI. Adjusted *p*-values were calculated using the Bonferroni correction for multiple comparisons within the table.

**Supplementary Table S3.** Pearson Correlations Between Weekly Active Gameplay and Changes in Body Composition and Grip Strength

| **Outcome** | **r** | ***p*-value** | **Adjusted *p*-value** |
| --- | --- | --- | --- |
| Δ Fat Mass (kg) | 0.253 | 0.327 | 1.000 |
| Δ Fat-Free Mass (kg) | -0.267 | 0.300 | 1.000 |
| Δ Right Grip Strength (kg) | 0.269 | 0.296 | 1.000 |
| Δ Left Grip Strength | 0.336 | 0.187 | 1.000 |
| Δ Total Grip Strength | 0.365 | 0.150 | 0.898 |

Pearson correlation coefficients (*r*) describe the association between average weekly active gameplay minutes and changes in physiological outcomes from pre- to post-intervention. Adjusted *p*-values were calculated using the Bonferroni correction for multiple comparisons. None of the associations remained statistically significant following adjustment.

**(A)**	**(B)**

**(C)	(D)**

 **(E)**

**Supplementary Figure S1.** Scatterplots illustrating dose–response relationships between active gameplay exposure (minutes/week) and changes in physiological outcomes over the 8-week intervention. (A) Δ Grip Right; (B) Δ Grip Left; (C) Δ Grip Total; (D) Δ Fat Mass (FM); (E) Δ Fat-Free Mass (FFM). Each point represents an individual participant. Lines represent univariate Pearson correlations. No associations remained statistically significant after correction for multiple comparisons.
